# Deficiency of C-reactive protein or human C-reactive protein transgenic treatment aggravates influenza A infection in mice

**DOI:** 10.3389/fimmu.2022.1028458

**Published:** 2022-10-06

**Authors:** Zhuohan Zhang, Yongjun Gao, Li Li, Junhao Luo, Rongbao Gao

**Affiliations:** ^1^ National Health Commission of People's Republic of China (NHC) Key Laboratory of Biosafety, National Institute for Viral Disease Control and Prevention, Chinese Center for Disease Control and Prevention, Beijing, China; ^2^ National Health Commission of People's Republic of China (NHC) Key Laboratory of Medical Virology and Viral Diseases, National Institute for Viral Disease Control and Prevention, Chinese Center for Disease Control and Prevention, Beijing, China

**Keywords:** influenza, C-reactive protein, pneumonia, immune response, immune checkpoint

## Abstract

C-reactive protein (CRP) has been shown to be a potential candidate target in the immunotherapy of severe influenza A infection. However, it is unclear on the pathogenesis associated with CRP in influenza infections. Here, we used influenza A H1N1 CA04 to infect human CRP transgenic mice (KI), CRP knockout mice (KO), and wild-type mice (WT), respectively, and compared the viral pathogenicity and associated immune response in those mice. The results showed that CA04 infection resulted in 100%, 80%, and 60% death in KO, KI, and WT mice, respectively. Compared to WT mice, CA04 infection resulted in higher TCID50 in lungs on day 3 after infection but lowered HI antibody titers in sera of survivors on day 21 after infection in KI mice. ELISA assay showed that IFN-**γ** concentration was significantly increased in sera of WT, KI, or KO mice on day 7 after infection, and IL-17 was remarkably increased in sera of WT mice but decreased in sera of KI mice while no significant change in sera of KO mice on day 3 or 7 after infection. Quantitative RT-PCR showed that the relative expression levels of immune checkpoint CTLA-4, LAIR-1, GITR, BTLA, TIM-3, or PD-1 mRNA in the lung presented decreased levels on day 3 or 7 after infection in KI or KO mice. The correlation analysis showed that mRNA expression levels of the 6 molecules positively correlated with viral TICD50 in WT mice but negatively correlated with viral TCID50 in KI or KO mice. However, only LAIR-1 presented a significant correlation in each lung tissue of WT, KI, or KO mice with CA07 infection statistically. IHC results showed that LAIR-1 positive cells could be found in WT, KO, or KI mice lung tissues with CA04 infection, and the positive cells were mainly distributed in an inflammatory dense area. Our results suggested that deficiency of CRP or human CRP **t**ransgenic treatment aggravates influenza A virus infection in mice. CRP is a double sword in immune regulation of influenza infection in which IL-17 and immune checkpoint may be involved.

## Introduction

The influenza virus that causes annually recurrent acute respiratory disease in humans is responsible for a large proportion of morbidity and mortality (1). The WHO estimates that annual influenza epidemics result in ~1 billion infections, 3-5 million cases of severe illness, and 290,000-650,000 deaths (2). Severe disease and/or mortality in patients with influenza virus infection are generally due to virus-induced pneumonia or secondary bacterial superinfection (1). Primary viral pneumonia is characterized by high levels of viral replication in the lower respiratory tract accompanied by strong pro-inflammatory responses. Influenza-mediated alveolar epithelial cell injury is due to inherent viral pathogenicity and imbalanced host immune response triggered by the virus (3, 4).

C-reactive protein (CRP), a pentameric protein found in almost all organisms where the presence of CRP has been sought, is an inflammatory biomarker and an immune mediator (5, 6). It was first named because of its ability to precipitate C-polysaccharide from Streptococcus pneumoniae *in vivo* and became a protein expressed as a component of the acute phase response in humans and some other species (7). Growing studies have shown that CRP plays important roles in inflammatory processes and host responses to infection, including the complement pathway, apoptosis, phagocytosis, nitric oxide release, and the production of cytokines. Several studies suggested that CRP is a potential candidate target in the immunotherapy of severe influenza A infection (5, 8–10). However, to our knowledge, it is unclear on the pathogenesis associated with CRP on severe influenza infection.

In this study, to understand the pathogenesis associated with CRP on severe influenza infection, we used influenza A H1N1 virus to infect mice with human CRP transgenic treatment, mice with deficiency of CRP, and wild-type mice, respectively, and compared the viral pathogenicity and associated immune response in the three typed mice.

## Materials and methods

### Mice and infection

All animal studies were performed according to the guidelines approved by the Investigational Animal Care and Use Committee of the National Institute for Viral Diseases Control and Prevention of the China CDC and were conducted following the guidelines of the Council for Animal Care. The CRP knockout (KO), human CRP knock-in (KI), or wild-type (WT) C57BL/6J mice were purchased from Cyagen Biosciences (Suzhou, China). The KO or KI mice were detected by PCR, sequencing, and southern blotting to determine the knockout of mouse CRP or knock-in of human CRP on the sampled tail of each mouse. We performed a viral challenge by i.n. Inoculation of 1.5×10^4^ TCID50 of A/California/04/2009 (H1N1) to anesthetized 8- to 10-week aged KO, KI, or WT female mice in 50 μL PBS. After the mice were infected with the virus, their body weight was measured daily to observe the changes. If the mice lost over 25% of their initial body weight, they were humanely euthanized and necropsied.

### Viral titration

The influenza viruses used in this study were titrated by a TCID50 (50% tissue culture infectious dose) in MDCK cells. Briefly, 100 μl/well of MDCK cells (3 × 10^5^ cells/ml) were seeded one day before infection in 96-well microtiter plates. Serial semi-logarithmic dilutions of each virus or supernatants of mouse lung homogenates were made with Dulbecco modified Eagle medium containing 1% bovine serum albumin and 2 μg/ml TPCK-treated trypsin from 10^−2^ to 10^−7^. Each virus or sample’s dilution was added to MDCK cells (4 wells for each dilution, 100 μl/well). The cells were incubated for 72 h at 35°C. The contents of each well were tested for hemagglutination by incubating 50 μl of the tissue culture supernatant with 0.5% turkey erythrocytes. The TCID50 was calculated according to the Reed and Muench method. For mouse lung tissue processing, in brief, left lung tissues from each mouse were homogenized in 1 mL of phosphate-buffered saline (PBS) by the tissue lyser (Qiagen). The supernatant was sampled after centrifugation at 3000 rpm for 15 min at 4°C.

### Hemagglutination-inhibition (HI) assay

Prior to testing by the HI assay with turkey RBC, the serum samples were treated with 4-fold receptor destroying enzyme (RDE) dilutions at 37°C for 18 h, followed by incubation at 56°C for 30 min. The serum samples were titrated in 2-fold dilutions of PBS and tested at an initial dilution of 1:10. Virus was added at a concentration of 4 HAU/25 μL. After 1 hour, 50 μL of 1% turkey RBC was added.

### Histopathological and immunohistochemical staining

Routine hematoxylin and eosin staining was used for histopathology evaluation. For immunohistochemistry, 4μm deparaffinized formalin-fixed paraffin-embedded sections were stained with polyclonal antibody against LAIR-1 (51030-R119, Sinobilogical, China) by using a polymer-based colorimetric indirect peroxidase method (ZSBio, China).

### Cytokine IL-17 and IFN-γ assay

The concentration of IL-17 and IFN-**γ** in mice sera was determined using an enzyme-linked immunosorbent assay (ELISA) according to the manufacturer’s instructions (R&D system, USA). The serum samples were detected in 10-fold dilutions of PBS. The concentrations of IL-17 and IFN-**γ** were calculated through standard curves using the standard product (**Supplemental Figure 1**).

### RNA extraction and quantitative RT-PCR

RNA was extracted from mouse lung tissues using an animal tissue total RNA extraction kit (TIANGEN BIOTECH, China) per the kit’s protocol. To quantify the relative expression levels of immune checkpoint glucocorticoid-induced TNF receptor family-related protein (GITR), B- and T-lymphocyte attenuator (BTLA), T-cell immunoglobulin and mucin-3 (TIM-3), cytotoxic T lymphocyte-associated antigen-4 (CTLA-4), human leukocyte associated Ig-like receptor-1 (LAIR-1) or programmed death 1 (PD-1) mRNA in mice lung tissues, a quantitative real-time RT-PCR was performed by QuantiFast SYBR Green RT-PCR Kit (Life Science Technologies, USA) on a real-time PCR detection system (Agilent Technologies Inc., Santa Clara, CA). The housekeeping gene GAPDH was used as the internal control. The specific primer sets were used as follows: GITR forward: GCCAGACGCTACAAGACT, GITR reverse: ATCGTAACTCACCGCTCT; BTLA forward: GTGACTTGGTGTAAGCACAATGGAA, BTLA reverse: TACGACCCGTTATCACTGAGATGTA; TIM-3 forward: AACCCTGCGAAAGGCAAACTT, TIM-3 reverse: GGTGACGACTGTCCTCCCAAA; CTLA-4 forward: AACCTTCAGTGGTGTTGGCTAG, CTLA-4 reverse: CCTCAGTCATTTGGTCATTTGT; LAIR-1 forward: TTGTCTTTCCGCCCTTCTGTTCTG, LAIR-1 reverse: CTGCTGCTGTCTTTTGTTGTTTGG; PD-1 forward: TATAACCTTGACGCAAACCA, PD-1 reverse: CTTGCTCATTTCAGAGTCCT; GAPDH reverse: ATGGGAGTTGTTTTCTTG, GAPDH forward: CTCGTCTTCTGTCATCTCTGCTG. The relative expression level was displayed by ΔCt in previous studies (11, 12).

### Statistical analysis

The mouse survival curve analysis was performed using Fischer’s exact test. Mouse body weight changes, viral TCID50 in mouse lung tissue, sera HI antibody titers, sera concertation of cytokines, and mRNA expression levels of immune checkpoints in mouse lung tissues were observed using unpaired t-tests for significant differences. The correlations between mRNA levels of checkpoint and sera levels of cytokines were analyzed by Pearson’s correlation method. Differences were considered significant at *P* <.05 with a two-tailed test. All analyses were performed using Instat software (Vision 5.0; GraphPad Prism).

## Results

### The impact of CRP on pathogenicity of influenza virus in mice

**Figure 1:**
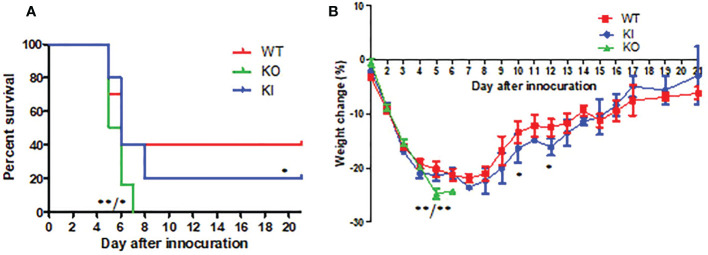
The survival rate and body weight changes in WT, KO, or KI mice with CA07 infection. **(A)** Kaplan-Meier survival curves were recorded. n=10 mice for each group. *P <0.05, **P <0.01 (log-rank test) when comparing the WT or KI mice. **(B)** Body weight loss was recorded for all survived mice until 21 days post-infection. *P<0.05, **P <0.01 (two-tailed *t*-test) when comparing the WT or KI mice.

**Figure 2:**
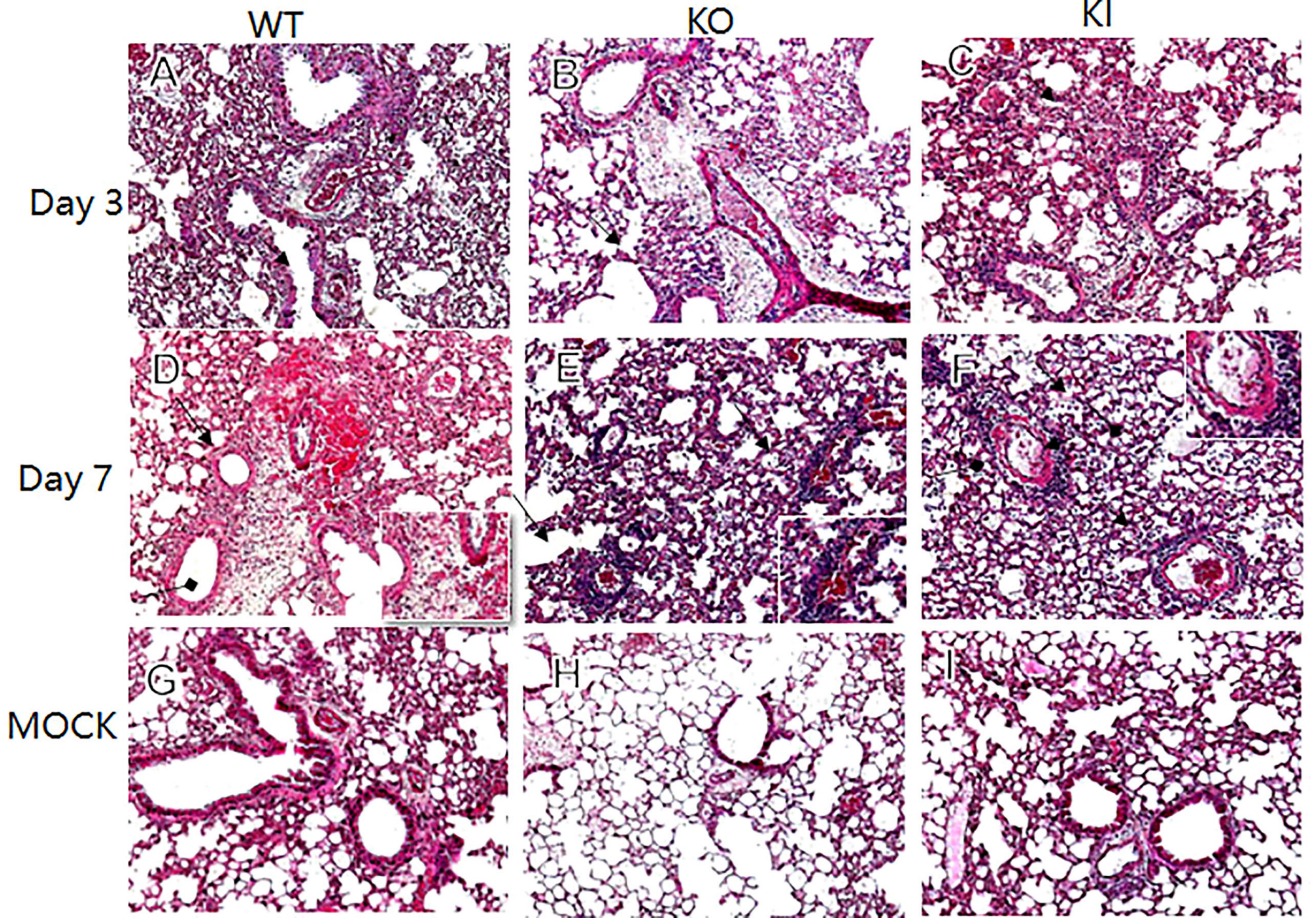
Histopathological damage in lung tissues of WT, KO or KI mice infected with CA04. Representative lung histopathology of CA04 or PBS challenged WT **(A, D, G)**, KO **(B, E, H)** or KI **(C, F, I)** mice on day 3 or 7 after infection. The lungs of PBS inoculated WT **(G)**, KO **(H)** or KI **(I)** mice were set as MOCK. The infiltration of inflammatory cells (black arrow) and hyaline membrane formation (black square arrow) were presented in lung sections. Original magnification: ×10.

CA04 infection resulted in 60% (n=10), 80% (n=10), or 100% (n=10) fatality in WT, KI, or KO mice respectively. The body weight changes suggested that the body weight loss of KO mice was higher than one of WT or KI mice after day 4 of infection, and the body weight loss of KI mice presented higher than one of the WT mice on day 7 after infection till to day 14 after infection (**Figure 1**). Furthermore, histopathological changes were observed on WT, KI, or KO mice with CA07 infection lung tissues. The results showed that, compared to MOCK, the CA04 resulted in typical histopathological damages, including infiltration of inflammatory cells and congestion and edema of alveoli and bronchus in the lungs of WT mice as well as in the lungs of KO or KI mice (**Figure 2**). On day 3 after infection, CA04 raised focal damages with scatted bronchopneumonia in WT, KO, or KI mice.

In comparison, the infiltration of inflammatory cells presented more in WT or KI mice than in KO mice (**Figures 2A–C**). On day 7 after infection, defuse alveolar damages were observed in the lungs of WT, KO, or KI mice. In comparison, the damages were more serious and extensive in the lungs of KO or KI mice than those of WT mice (**Figures 2D–F**). Taken together, the results suggested that deficiency of CRP or human CRP transgenic treatment enhanced the pathogenicity of influenza virus in mice.

### The impact of CRP on viral replication and antibody response in mice with influenza virus infection

**Figure 3:**
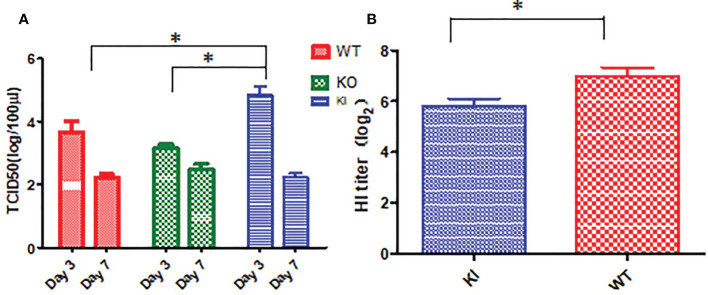
Viral load and HI antibody titer in mice with CA04 infection. **(A)** The viral TCID50 in lung tissues of CA04 infected WT, KI, or KO mice on day3 or 7 after infection, n=5 mice per group (mean ± SEM). **(B)** HI antibody titers in sera of CA04 infected WT or KI mice on day21 or 7 after infection. Unpaired t-tests were performed to assess statistical significance, *P <0.05, (two-tailed).

Viral titration showed that the viral TCID50 presented a significantly higher level in the lungs of KI mice than WT or KO mice on day 3 after infection. In contrast, no significant difference was observed between them, although viral TCID50 presented a higher level in the lungs of KO mice than KI or WT mice on day 7 after infection (**Figure 3A**). The HI assay showed that the sera HI antibody titer against CA07 presented a much higher level in WT survivors than in KI survivors (**Figure 3B**). The results suggested that CRP was related to the viral clearance and antibody response in influenza infection.

### The impact of CRP on levels of cytokine IFN-γ and IL-17

**Figure 4:**
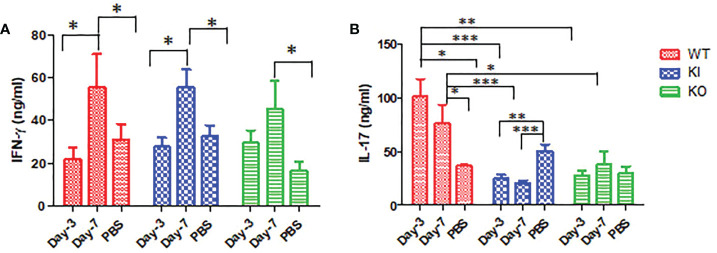
IFN-γ and IL-17 levels in WT, KO, or KI mice sera. **(A)** The concentration of IFN-γ tested by ELISA in sera of WT, KO, or KI mice infected with CA04 on day 3 or 7 after infection, PBS inoculated mice were set as mock control. **(B)** The concentration of IL-17 tested by ELISA in sera of WT, KO, or KI mice infected with CA04 on day 3 or 7 after infection, PBS inoculated mice were set as mock control. Unpaired t-tests were performed to assess statistical significance, *P <0.05, **P <0.01, ***P <0.001 (two-tailed)..

As shown in **Figure 4**, the ELISA assay showed that IFN-**γ** concentration was significantly increased in sera of WT, KI, or KO mice on day 7 after infection, and no significant difference was observed between them. However, compared to PBS inoculated mice, IL-17 was remarkably increased in sera of WT mice with CA04 infection but decreased in sera of KI mice with CA04 infection on day 3 or 7 after infection, while there was no significant change in sera of KO mice infected with CA04 on day 3 or 7 after infection. The results suggested that deficiency of CRP or human CRP transgenic treatment decreased IL-17 immune response in influenza infection.

### The impact of CRP on immune checkpoint mRNA expression of infected mice

**Figure 5:**
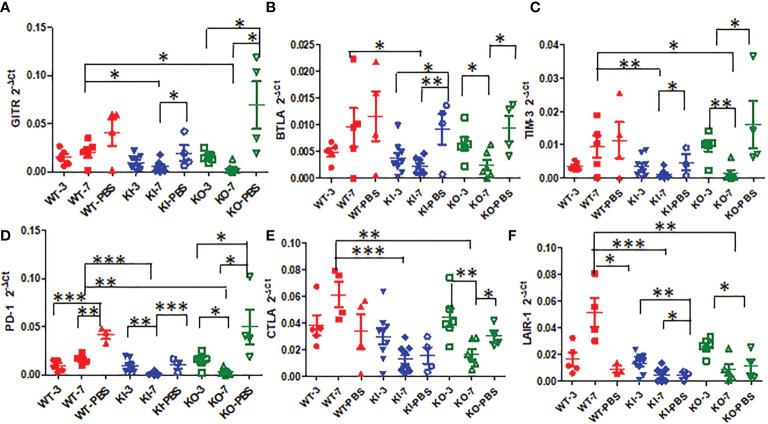
The relative quantification level of checkpoint mRNA GITR **(A)**, BTLA **(B)**, TIM-3**(C)**, CTLA-4 **(D)**, PD-1 **(E)**, or LAIR-1 **(F)** in lung tissues of WT, KO, or KI mice with CA04 infection on day 3 or 7 after infection. PBS inoculated mice were set as mock control. Unpaired *t*-tests were performed to assess statistical significance, *P <0.05, **P <0.01, ***P <0.001 (two-tailed).

**Figure 6:**
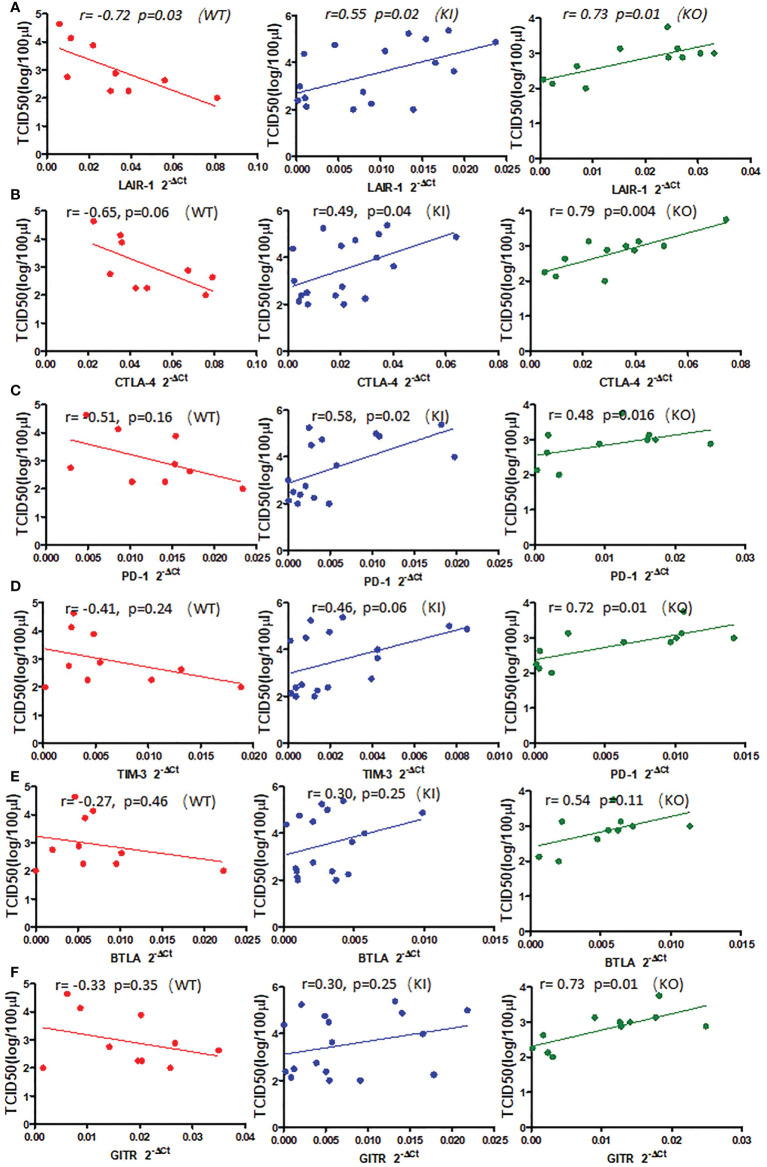
The correlation between relative quantification level of checkpoint mRNA LAIR-1 **(A)**, CTLA-4 **(B)**, PD-1 **(C)**, TIM-3 **(D)**, BTLA **(E)**, GITR **(F)**, and viral TCID50 in lung tissue of WT, KO, or KI mice infected with CA04 on day 3 or 7 after infection. Pearson correlation analysis was performed.

**Figure 7:**
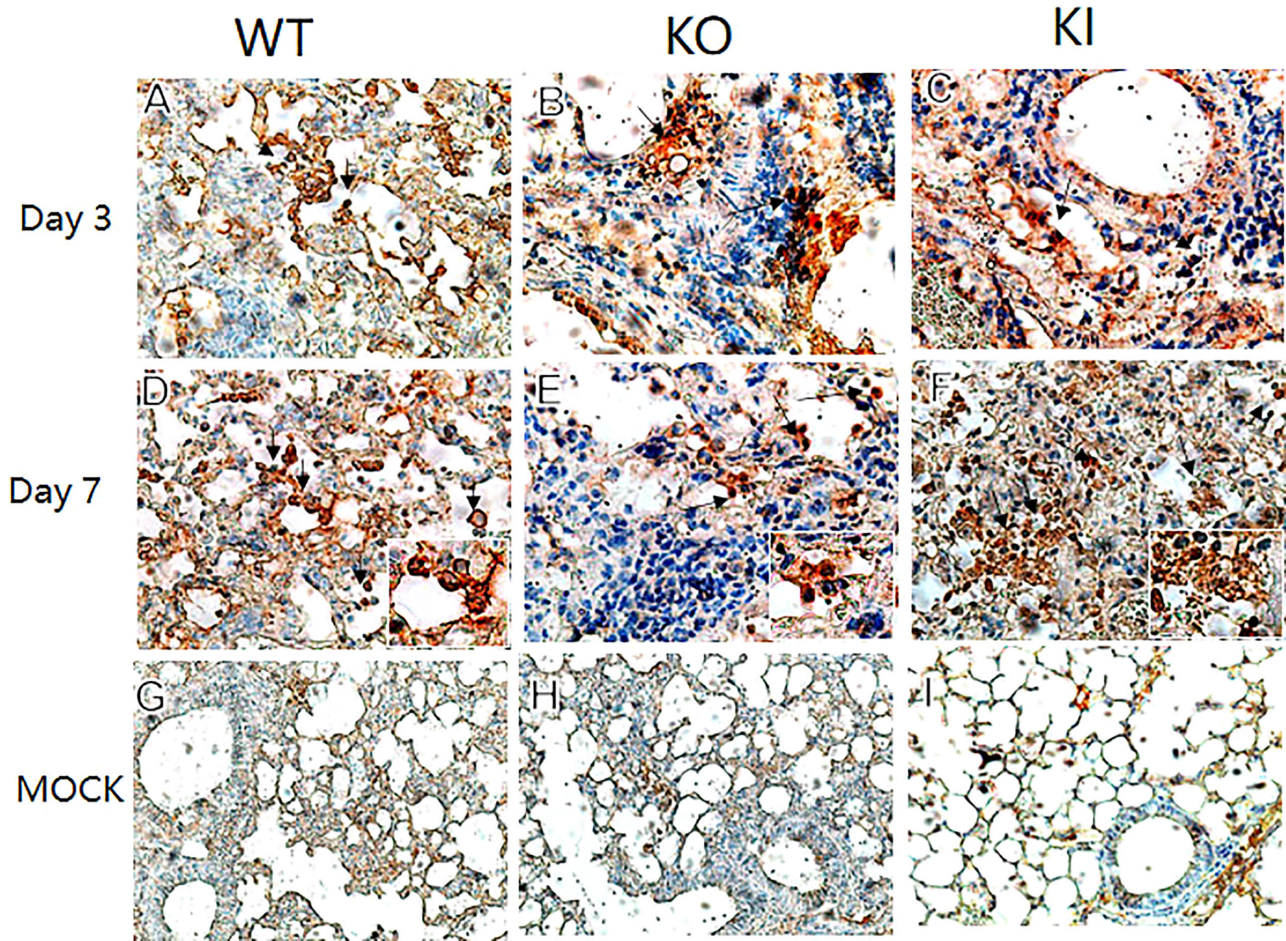
LAIR-1 immunopathology of lung sections from WT, KO, or KI mice. Immunohistochemistry for LAIR-1 + pulmonary cells (black arrows) in representative lung sections of WT, KO, or KI mice infected with CA04 on day 3 **(A–C)** or 7 after infection **(D–F)**. PBS inoculated mice were set as mock control **(G–I)**. Original magnification: ×40 **(A–F)** or ×20 **(G–I)**.

To observe the correlation of CRP with the local immune response of lung in mice to influenza infection, we quantified the relative expression levels of 6 immune checkpoint mRNAs in lung tissues of mice, including GITR, BTLA, TIM-3, PD-1, CTLA-4, and LAIR-1. The results showed that compared to PBS inoculated mice, relative expression levels of GITR, BTLA, TIM-3, and PD-1 mRNA were significantly decreased on day 3 or 7 after infection in KI or KO mice with CA07 infection but not in WT mice with CA07 infection, and their levels were significantly higher in WT mice than in KI or KO mice (**Figures 5A–D**). Whereas the relative expression levels of CTLA-4 and LAIR-1 were increased on day 3 or 7 after infection in WT, KI, and KO mice, and their levels were significantly higher in WT mice than in KI or KO mice on day 7 after infection (**Figures 5E, F**). In addition, the correlation analysis showed that mRNA expression levels of the 6 molecules presented a respectively positive correlation with viral TICD50 in WT mice but a negative correlation with viral TCID50 in KI or KO mice (**Figure 6**). However, only LAIR-1 presented a significant correlation in each lung tissues of WT, KI, or KO mice with CA07 infection statistically (**Figure 6A**). In addition, IHC results showed that LAIR-1 positive cells could be seen in lung tissues of WT, KO, or KI mice with CA04 infection, and the positive cells were mainly distributed in an inflammatory dense area. Given the comparison, more stained cells were seen on day 7 after infection than on day 3 after infection and in WT and KI mice than in KO mice on day 7 after infection (**Figure 7**).

## Discussion

The modulation of host factors involved in regulating viral replication and/or injury or tissue recovery has been demonstrated to be a potential strategy against viral diseases, including influenza (13, 14). Whereas, CRP has been considered a potential therapeutic target for inflammatory diseases, including infections, since CRP bound to a multivalent ligand can efficiently initiate the assembly of a C3 convertase through the classical pathway and thus decorate the surface of the ligand with opsonic complement fragments (6, 15, 16). However, the detailed response pathway of CRP to the disease is still unknown, although CRP is related to the outcome of severe influenza disease and joined in the mediation of immunopathological lesions (8, 17, 18).

Our data demonstrated that deficiency of CRP or human CRP transgenic treatment aggravated influenza A virus infection in mice, and deficiency of CRP resulted in a much more severer outcome than human CRP transgenic treatment. Human CRP transgenic mice have been demonstrated to be a good model for studying the *in vivo* function of the protein (19) and have been used to study infectious diseases (6, 20–25). Transgenic or passively administered human CRP was protective against lethal bacterial infection in transgenic mice (6). In contrast, increasing studies have shown that excessively high CRP level was a risk factor for virus infection’s severity or fatal outcome, including influenza (8, 17, 26–28). Studies have demonstrated that the antiviral immune response represents a balancing act between the elimination of the virus and immune-mediated pulmonary injury (29). Our previous study showed that CRP joined in mediating immunopathological lesions in severe influenza (8). Hence, our results here indicated that CRP might play an important role in the immune balance of influenza infection, and the role may be a double-edged sword in influenza infection, overexpression or deficiency of CRP would be a disadvantage to the infection. Besides survival rate and body weight loss, our results showed that human CRP transgenic treatment or deficiency of CRP resulted in more serious and extensive damage to the lung in mice with influenza A infection on day 7 after infection, and human CRP transgenic treatment increased the viral load in the lung of mice with influenza A infection on day 3 after infection but decreased the HI antibody titer in survivor on day 21 after infection. The results indicated that deficiency of CRP or human CRP transgenic treatment impacted the immune response associated with tissue damage, viral clearance, and/or antibody production.

Our results also suggested that deficiency of CRP or human CRP transgenic treatment decreased or blocked the immune response of IL-17 in influenza A infection. Studies showed that IL-17 plays a critical role in mediating the recruitment of B cells to the site of pulmonary influenza virus infection in mice (29) and suggested that anti-IL-17A or anti-IFN-**γ** treatment attenuated the severity of immunopathology by influenza virus (30, 31). However, studies also suggested that IL-17 plays a crucial role in enhancing effective antiviral immune responses, including the maintenance of tissue integrity and the generation of protective immune responses to infectious microorganisms, especially at epithelial barrier sites (32–34). Our results showed that both deficiencies of CRP and human CRP transgenic treatment decreased IL-17 response but had no influence on IFN-**γ** response in mice with influenza A infection, indicating that IL-17 antiviral response was deficient or decreased in influenza A infection in mice with deficiency of CRP or human CRP transgenic treatment or.

We further analyzed the impact of CRP on the expression of immune checkpoint molecules in lung tissues of mice with influenza A infection because several immune checkpoint molecules have been demonstrated to be crucial for maintaining self-tolerance and for modulating the length and magnitude of effector immune responses in peripheral tissues to minimize tissue damage (35–37). In addition, studies of the interplay between immune activation and suppression have shown an important role for immune checkpoint molecules in the pathogenesis of infectious diseases (37). In this study, our results showed that, compared to WT mice, influenza A infection resulted in decreased expression of checkpoint molecules GITR, BTLA, TIM-3, PD-1, CTLA-4, and LAIR-1 in lung tissues of KI or KO mice on day 7 after infection, and expression levels of these molecules presented a positive correlation with viral TICD50 in lungs of WT mice but negative correlation with TCID50 in lungs of KI or KO mice although the significant correlation was not observed in all them (**Figure 5** and **Supplemental Figure 1**). And the results showed the only LAIR-1 presented significant correlation with viral TCID50 in each infection of the three typed mice. The IHC stains also showed that LAIR-1 positive cells were mainly distributed in the inflammatory dense area of lung tissues in WT, KO, or KI mice with CA04 infection. Studies showed that LAIR-1 plays a role in regulating immune cells (38) and limits neutrophilic airway inflammation as a functional inhibitory receptor on airway-infiltrated neutrophils (39, 40). The results indicated that deficiency of CRP or human CRP transgenic treatment impacted the balance of immune regulation by immune checkpoint molecules. In contrast, the delicate immune balance is a key factor in maintaining normal immune responses such as viral clearance (41), tissue tolerance, antibody responses, and tissue repairment (29, 42, 43).

In summary, we observed the impact of human CRP transgenic treatment and deficiency of CRP on the pathogenicity of influenza A virus in mice and analyzed immune factors associated with innate immune regulation in those mice. Our results showed that both deficiencies of CRP and human CRP transgenic treatment aggravated influenza A infection in mice, and the aggravation may be owed to imbalance immune regulation, including decreased antibody response, IL-17 levels, and/or expression of several immune checkpoint molecules.

## Data availability statement

The original contributions presented in the study are included in the article/**Supplementary Material**. Further inquiries can be directed to the corresponding author.

## Ethics statement

The animal study was reviewed and approved by Investigational Animal Care and Use Committee of the National Institute for Viral Diseases Control and Prevention of the China CDC.

## Author contributions

RG designed the study and wrote the report. ZZ, YG, and LL performed the experiments. RG, ZZ, and YG joined in the data analysis. All authors contributed to the review and revision of the manuscript and have read and approved the final version.

## Funding

This work was supported by the National Natural Scientific Foundation of China (grant number 81971946) and the National Key R&D Program of China (grant number 2019YFC1605001). The contents of this article are solely the responsibility of the authors and do not necessarily represent the views of China CDC and other organizations.

## Conflict of interest

The authors declare that the research was conducted in the absence of any commercial or financial relationships that could be construed as a potential conflict of interest.

## Publisher’s note

All claims expressed in this article are solely those of the authors and do not necessarily represent those of their affiliated organizations, or those of the publisher, the editors and the reviewers. Any product that may be evaluated in this article, or claim that may be made by its manufacturer, is not guaranteed or endorsed by the publisher.

## Supplementary material

Supplementary Figure 1 The ELISA standard curves of IL-17 **(A)** and IFN-**γ (B)**.
