# Supplementary figures and images for: Deficiency of C-reactive protein or human C-reactive protein transgenic treatment aggravates influenza A infection in mice
Source: Front Immunol. 2022 Oct 6;13:1028458. doi: 10.3389/fimmu.2022.1028458 (PMC9584053; doi:10.3389/fimmu.2022.1028458)

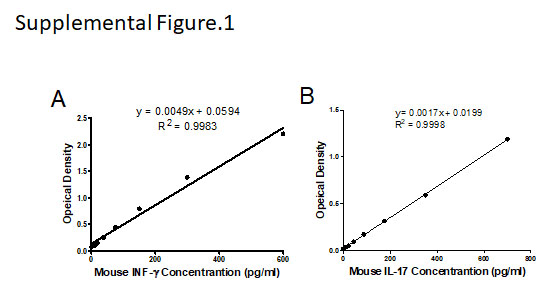

Supplement: Supplementary Figure 1 — The ELISA standard curves of IL-17 (A) and IFN-γ (B). [file Image_1.jpeg]
